# Risk factors of retinal vein occlusion in East Asia: a meta-analysis

**DOI:** 10.3389/fopht.2025.1545602

**Published:** 2025-03-24

**Authors:** Xinyue Qiu, Ziman Jiao, Yuxin Liu, Yunhao Zhou, Haiyu Li, Xin Chen, Guanghui Liu

**Affiliations:** ^1^ Department of Ophthalmology, Affiliated People's Hospital (Fujian Provincial People's Hospital), Fujian University of Traditional Chinese Medicine, Fuzhou, China; ^2^ Eye Institute of Integrated Chinese and Western Medicine, Fujian University of Traditional Chinese Medicine, Fuzhou, China; ^3^ Department of Bioengineering, College of Biological Science and Biotechnology, Fuzhou University, Fuzhou, China

**Keywords:** retinal vein occlusion, East Asia, risk factors, meta-analysis, hypertension

## Abstract

**Objective:**

With the intention of developing a more targeted control strategy for retinal vein occlusion (RVO) in East Asian populations, a meta-analysis was conducted to evaluate the risk factors associated with RVO in this region.

**Methods:**

PubMed, Web of Science, Cochrane Library, CNKI, Wanfang and VIP databases were searched for studies that reported risk factors of RVO in East Asia, published from the establishment of the database to May 2024. To further filter the articles, Newcastle-Ottawa Scale (NOS) evaluation method was utilized to assess the quality of selected articles. After valid data were extracted, Meta-analysis was performed by Review Manager software.

**Results:**

A total of 21 literatures were included, including 27561 cases in the RVO group (Case group) and 514578 cases in the non-retinal vein occlusion (NRVO) group (Control group). Results of meta-analysis showed that chronic kidney disease [odds ratio (OR)=4.14, 95% confidence interval (CI): (1.86%, 9.24%)], hypertension [OR=4.11, 95% CI: (3.09%, 5.48%)], hyperlipidemia [OR=3.45, 95%CI: (2.32%, 5.12%)], diabetes mellitus [OR=3.00, 95%CI: (1.88%, 4.80%)], homocysteine [OR=0.87, 95%CI: (0.59%, 1.15%)], have statistically significant differences between the RVO group and the NRVO group(*P*<0.05).

**Conclusion:**

The occurrence of RVO is closely related to its risk factors, such as chronic kidney disease, hypertension, hyperlipidemia, diabetes mellitus and high homocysteine. In the process of diagnosis and treatment of RVO, doctors should focus on the above risk factors to prevent the occurrence of the disease.

## 1 Background

Retinal vein occlusion (RVO) is a common group of retinal vascular diseases that clinically impair visual function. It is the second most prevalent retinal disorder, following diabetic retinopathy. By 2040, the number of RVO patients in Asia is projected to reach 21 million (1).

Depending on the site of the occlusion, RVO can be classified into central retinal vein occlusion (CRVO) or branch retinal vein occlusion (BRVO), with hemi-central retinal vein occlusion (HRVO) also classified under BRVO. Based on the presence or absence of ischemia, RVO can be further divided into ischemic and non-ischemic forms, with the prevalence ratio of ischemic to non-ischemic RVO being approximately 3:7 (2). However, with the course of the disease, some non-ischemic RVO can be transformed into ischemic RVO. Visual acuity in patients with RVO typically deteriorates rapidly. The main causes of visual acuity decrease are the complications of the disease, such as macular edema, neovascular glaucoma, vitreous hemorrhage, retinal detachment, etc.

Current treatments for RVO include laser therapy, intravitreal corticosteroid injections, vascular endothelial growth factor (VEGF) inhibitors and glucocorticoid therapy (including slow-release agents). Anti-VEGF therapy has become the first-line treatment for RVO with macular edema (3). However, there are also many patients who have received these treatments, and macular edema has not subsided, or some patients have a poor visual prognosis although the degree of macular edema has improved (4). Therefore, early prevention of RVO, identification of risk factors, and management of these risk factors are key areas of focus in ophthalmological research.

With the improvement of living standards, increasing work pressure, and changes in dietary and sleep habits, the age of onset for RVO is decreasing (5). Additionally, RVO is characterized by a complex and varied etiology. Studies have demonstrated a close association between RVO incidence and conditions such as hypertension, diabetes mellitus (DM), hyperlipidemia, and elevated homocysteine (Hcy) levels. Furthermore, RVO incidence is also linked to other systemic and ocular factors, including axial length, intraocular pressure (6).

Further research is needed to elucidate the specific risk factors for RVO across diverse populations, considering genetic, environmental, and lifestyle variations among ethnic groups. In East Asia, which has distinct demographic, genetic, and environmental traits compared to Western populations, understanding of the risk factors for RVO is essential for devising effective local prevention and management strategies.

East Asia, including countries such as China (including Hong Kong, Macao and Taiwan), Japan, North Korea, South Korea and Mongolia, is distinguished by its unique genetic diversity, cultural practices, and environmental exposures, all of which may influence the risk factors for RVO in the region (7). Therefore, a focused investigation of the risk factors for RVO in East Asia is necessary to address the specific needs of this population. Although there have been individual studies examining various risk factors for RVO in East Asia, there is a lack of comprehensive synthesis of the available evidence through meta-analysis methods.

The primary objective of this meta-analysis is to systematically review and analyze the literature to identify and assess RVO risk factors in East Asia, thereby providing insights for early prevention and treatment strategies.

## 2 Methods

Literature collection: in this study, PubMed, Web of Science, Cochrane Library, CNKI, Wanfang and VIP databases were used to search relevant reports on RVO risk factors in East Asia from the establishment of the database to May 2024. Five countries (China, Japan, North Korea, and South Korea, Mongolia) and 3 regions of China (Hong Kong, Macao, and Taiwan) were included. Search terms included “retinal vein obstruction; Retinal vein thrombosis; Retinal vein occlusion; Risk factors; Related factors; Influencing factors; Risk; Factor”. After combining subject terms and free-text terms for the computer search, additional manual searches were conducted to identify relevant literature.

Literature screening: Inclusion criteria: (1) case-control or cohort studies with RVO risk factors; (2) The subjects were East Asian individuals accurately diagnosed with RVO; (3) Studies providing original data for statistical analysis.

Exclusion criteria: (1) Cross-sectional studies lacking controls, which cannot establish causality; (2) Secondary studies, such as literature reviews; (3) Duplicate reports; (4) Studies from which data could not be obtained.

Data collection: General data (author, source, publication date, etc.) and subject characteristics (sample size, past medical history, distribution of risk factors, including systemic and hereditary diseases, etc.) were extracted. Quality assessment and data extraction of included studies were carried out one by one by two evaluators and cross-checked. Newcastle-Ottawa Scale (8) (NOS) (**Table 1**) was used to evaluate the quality of the retrieved literature. Quality evaluations with scores of 5 or higher were included in the meta-analysis. In case of any objection, the third member settle the dispute through arbitration.

**Table 1:** NOS rating sheet.

Column	Entry	Evaluation criteria
**Research object selection**	1. Is case identification appropriate (1 point)	(1) Appropriate, independent determination method or personnel; (2) Appropriate. Such as independent archival records; (3) Not described.
	2. Representativeness of cases (1 point)	(1) Continuous or representative series of cases; (2) Potential selection bias or undescribed.
	3. Choice of comparison (1 point)	(1) Control of the same population as the case; (2) The hospitalized patients in the same population as the case were the control; (3) Not described.
	4. Determination of comparison (1 point)	(1) No target disease more; (2) Not described.
**Comparability between groups**	Comparability of cases and controls was considered in design and statistical analysis (2 points)	(1) The study controlled for the most important confounding factors; (2) The study controlled for any other confounding factors.
**Exposure factor measurement**	1. Determination of exposure factors (1 score)	(1) Fixed archival records (e. g. surgical records); (2) The situation of using structured interview and not knowing the interviewer; (3) Interview was used but blind method was not implemented; (4) Not stated.
	2. The same method was used to determine whether the case and control group were dew factors (1 point)	(1) Yes; (2) No
	3. No response rate (1 point)	(1) The non-response rate of patients and pairs was the same; (2) Non-response rates were different between cases and controls and were not described.

Data analysis: meta-analysis was performed using RevMan 5.4 software (available at http://tech.cochrane.org/revman/download). Initially, the data were tested for heterogeneity. A fixed-effect model was selected for combined analysis if no significant statistical heterogeneity was detected (defined as *P* ≥ 0.1 and I² ≤ 50%). Conversely, a random-effects model was employed if statistical heterogeneity was present (defined as *P* < 0.1 and I² > 50%). In cases of substantial heterogeneity, subgroup analysis or sensitivity analysis was performed to identify the sources and causes of heterogeneity. A funnel plot was used to visually assess publication bias. Sensitivity analysis was conducted using the article exclusion method to evaluate the stability of the results. If the results remained unchanged after excluding individual studies, the findings were considered robust. For statistical analysis, the odds ratio (OR) was used as the effect size for categorical data, while the weighted mean difference (WMD) was used for continuous data. A 95% confidence interval (CI) was calculated for each effect size, and *P*<0.05 was considered statistically significant.

## 3 Results

### 3.1 Literature search results

**Figure 1:**
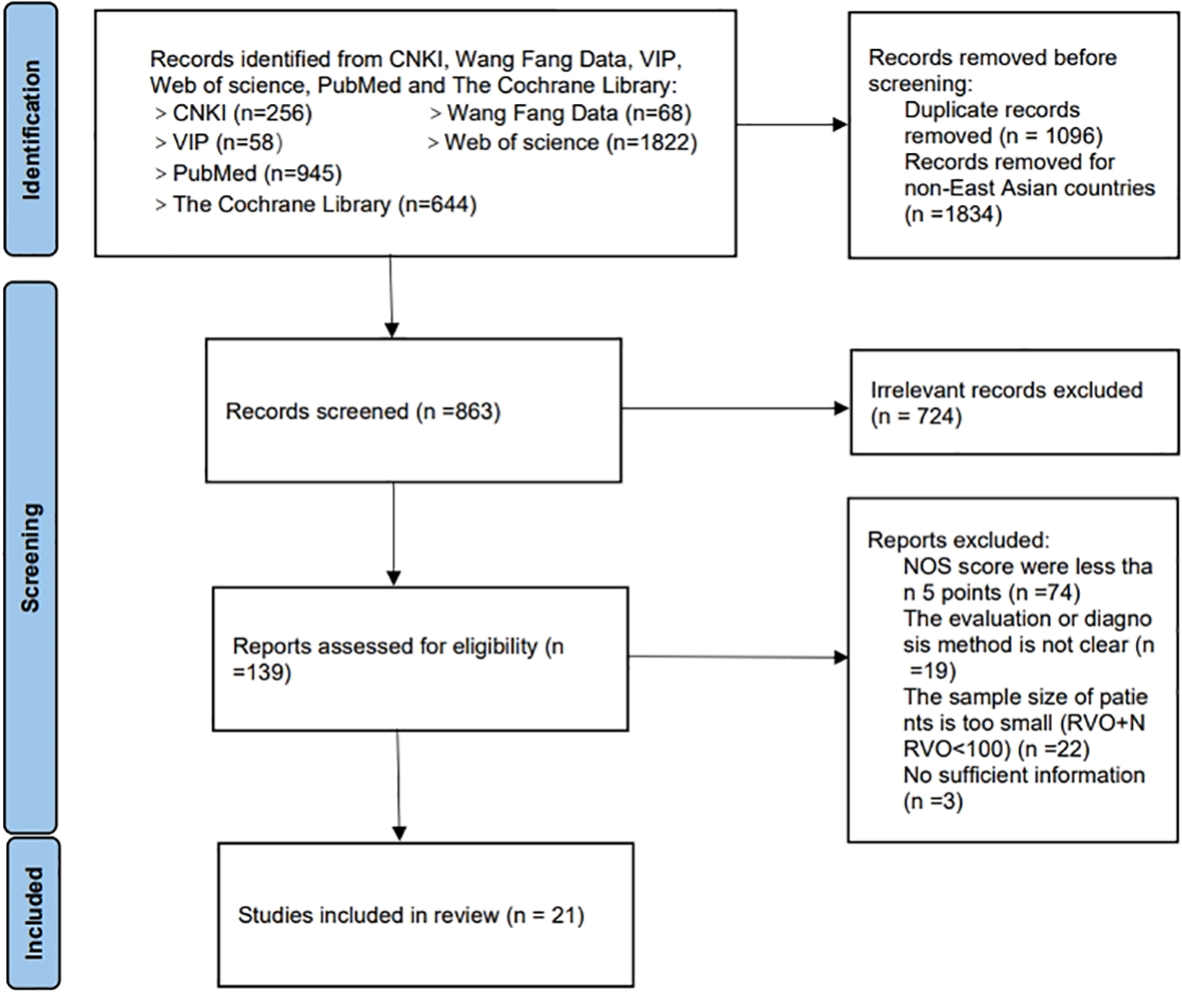
Literature screening flow chart.

A total of 3793 literatures were retrieved, 2930 literatures were excluded from East Asia, 724 literatures were excluded from title and abstract reading, 139 were preliminarily screened, 118 literatures were excluded from full text reading, and 21 were included in intensive re-screening. Details would be seen in **Figure 1**.

### 3.2 Basic characteristics of included studies and NOS scores

Among the 21 literatures included in this study, 3 from South Korea, 2 from Japan, 3 were from Taiwan of China and 13 from China mainland. In this study, risk factors including hypertension, DM, hyperlipidemia, high levels of lipoprotein a, high Hcy and so on were selected for systematic evaluation. Specific results were shown in **Table 2**.

**Table 2:** The basic characteristics and NOS score into literature.

Authors	Country/Area	Published time	Sample size	Median follow-up (months)	NOS score	Risk factors
Chia-Hsiang Shih (9)	Taiwan, China	2015	50427	120	7	①②③④⑤⑥
Rim, Tyler Hyungtaek (10)	South Korea	2014	10011	60	8	①②④⑤⑥
Chou, Kun-Ta (11)	Taiwan, China	2012	35634	120	7	①
Arakawa, Satoshi (12)	Japan	2011	1369	120	7	①④⑦
Yasuda, Miho (13)	Japan	2010	1774	1	7	①
Gao, Wei (14)	China	2006	172	11	6	①②③
Chen, Mei Ling (15)	China	2022	400	60	6	⑧⑪⑫
Li, Shuang Jun (16)	China	2021	370	72	6	⑬⑭;
Fan, Li Ming (17)	China	2017	258	48	6	①②
Hu, Yi Jun (18)	China	2017	158	62	6	①③⑧⑩⑮
Xu, Dong (19)	China	2020	138	30	6	①③⑧⑩⑪
Wu, Peng Chen (20)	China	2014	1774	12	6	①⑦
Duan, Xiao Li (21)	China	2017	8694	24	6	①③⑦⑧⑨⑩
Zhang, Peng (22)	China	2013	537	41	6	⑧⑪⑫
Zhang Xiaobo (23)	China	2017	140	12	7	①③⑧⑩
Wang Hongya (24)	China	2022	110	30	5	⑬⑭;
Ma Xiaomin (25)	China	2017	88	42	5	⑧
Dong, N (26).	China	2013	136	48	6	⑧
Ho, J. D (27).	Taiwan, China	2009	2450	24	7	①②③④
Park, H. L (28).	South Korea	2017	420536	132	8	①②③
Rim, T. H (29).	South Korea	2015	6105	108	7	①②④

(①Hypertension ②DM ③Hyperlipidemia ④Chronic kidney disease (CKD) ⑤Chronic liver disease(CLD) ⑥Cerebrovascular disease ⑦Age>60 ⑧Hcy ⑨BMI>25 ⑩Axis oculi<22mm ⑪Atherosclerosis of the carotid artery ⑫Low folate ⑬Neutrophil-to-lymphocyte ratio (NLR) ⑭;Platelet-to-lymphocyte ratio (PLR) ⑮Lipoprotein A).

### 3.3 Meta-analysis of risk factors for overall RVO

**Figure 2:**
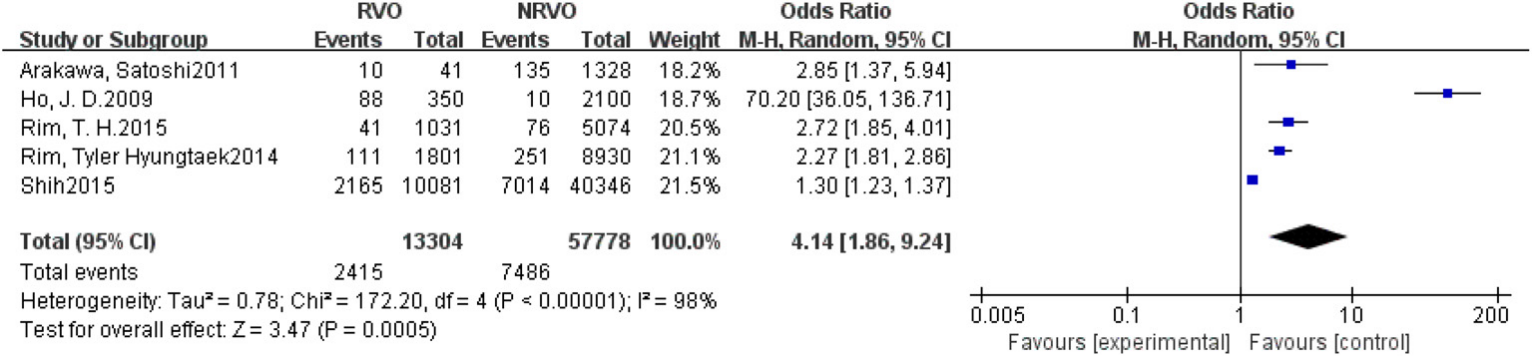
The forest plot between the RVO and CKD (OR, odds ratio; CI, confidence).

**Figure 3:**
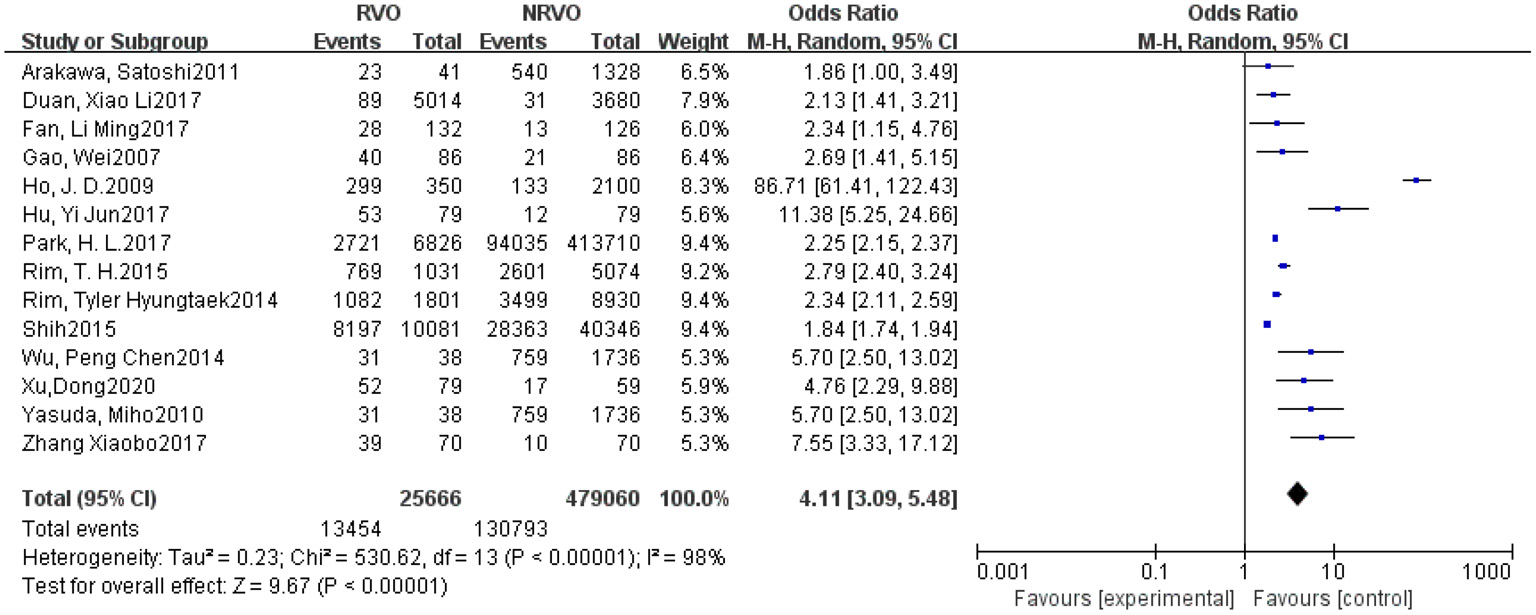
The forest plot between the RVO and hypertension (OR, odds ratio; CI, confidence).

**Figure 4:**
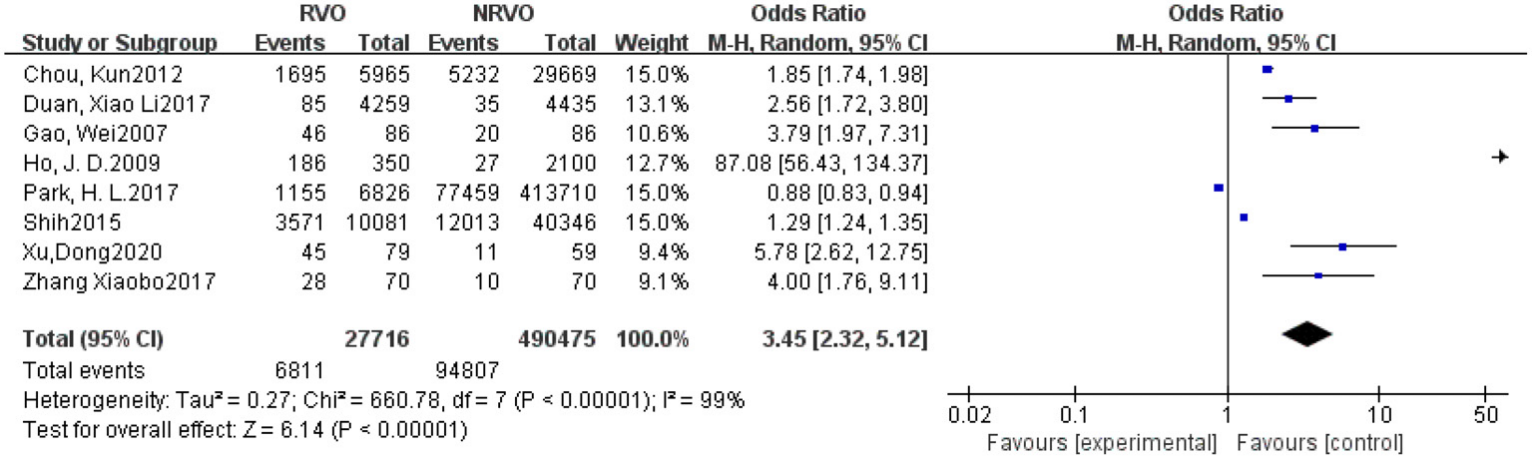
The forest plot between the RVO and hyperlipidemia (OR, odds ratio; CI, confidence).

**Figure 5:**
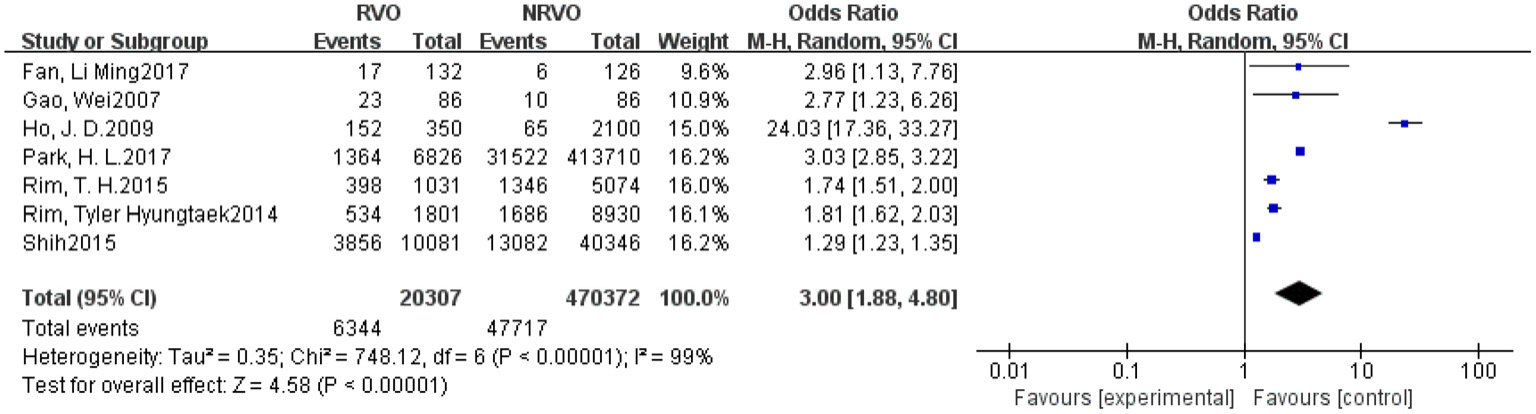
The forest plot between the RVO and DM (OR, odds ratio; CI, confidence).

**Figure 6:**
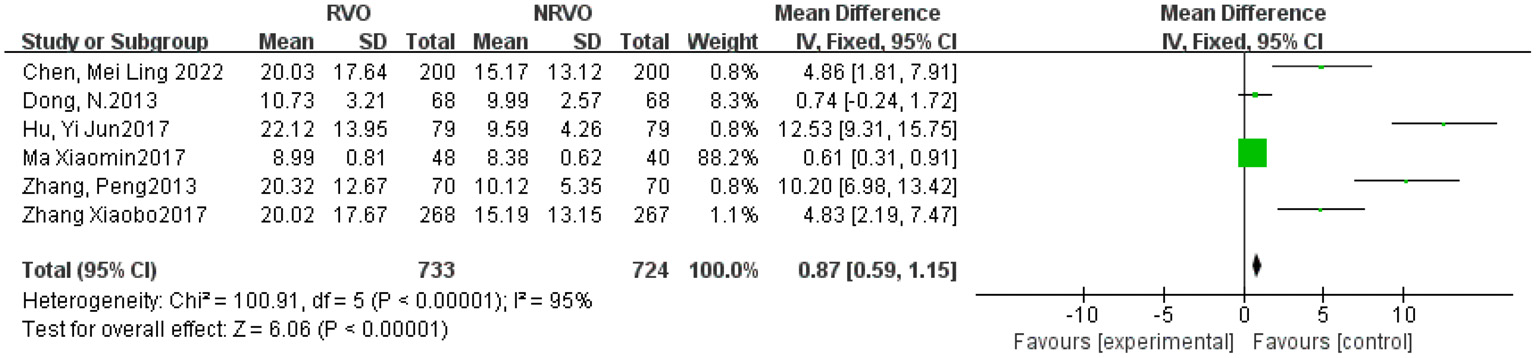
The forest plot between the RVO and Hcy (OR, odds ratio; CI, confidence).

**Figure 7:**
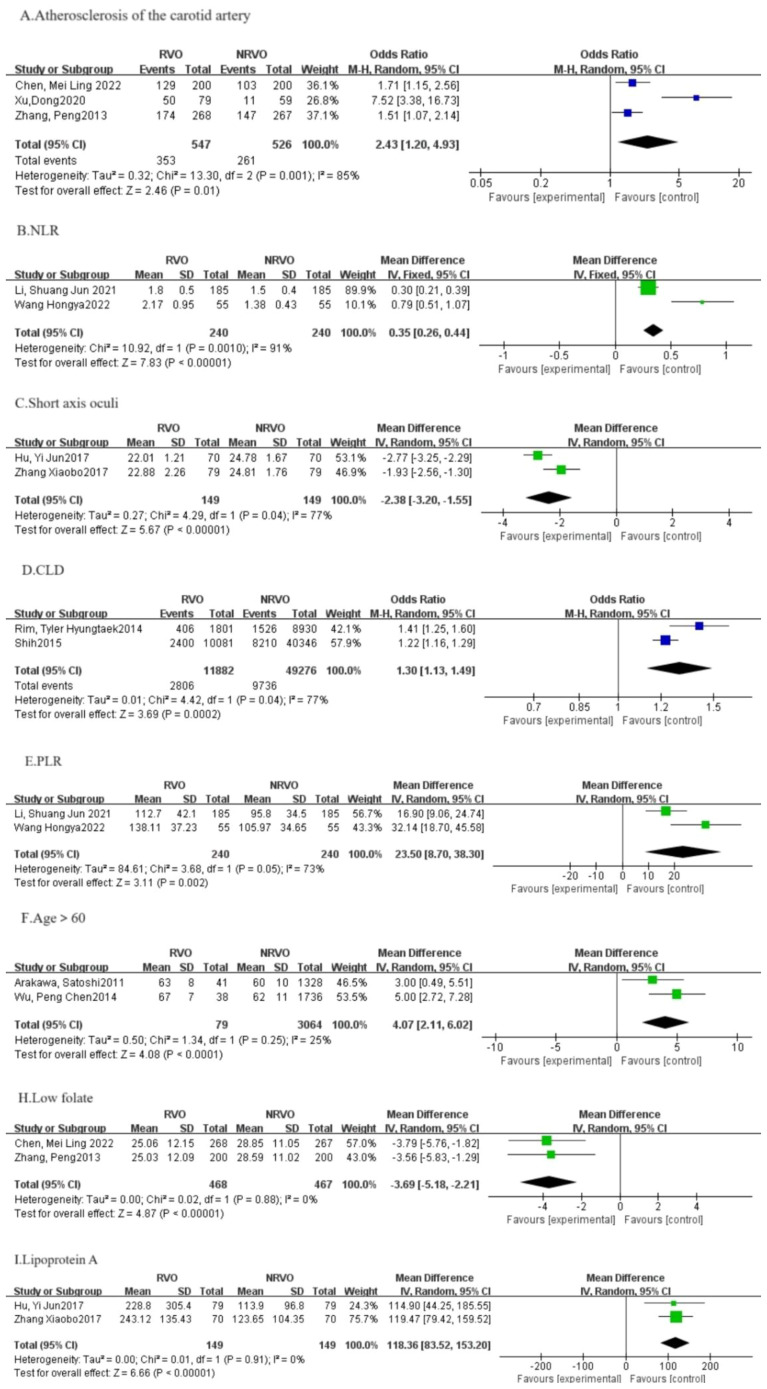
The forest plot between the RVO and the other factors (OR, odds ratio; CI, confidence).

Meta-analysis results showed that CKD, hypertension, hyperlipidemia, DM, and Hcy are risk factors for RVO (P<0.05), as shown in **Figures 2**–**7**.

## 4 Discussion

The precise etiology and pathogenesis of retinal vein occlusion (RVO) remain to be fully elucidated. However, it is widely hypothesized that RVO is associated with hemodynamic disturbances, including venous stasis, deterioration of blood vessel walls, and an increased predisposition to thrombosis (30). This study systematically reviewed reports on RVO risk factors in East Asia from the inception of the databases to 2024, comprehensively summarizing the correlation between exposure factors and RVO risk. The study concluded that RVO risk factors can be broadly categorized into systemic and ocular factors. Among the five major risk factors identified in this study, CKD, hypertension, hyperlipidemia, DM, and Hcy are all classified as systemic risk factors.

CKD, defined by kidney damage or an estimated glomerular filtration rate (eGFR) of less than 60 mL/min/1.73 m² sustained for over three months (31), emerges as a significant risk factor for RVO in our study.

Several factors may explain the association between RVO and CKD, with arteriosclerosis being a prominent contributor. Arteriosclerosis is a common feature in individuals with CKD and typically results from ongoing inflammation, fibrosis, hypertrophy, and calcification (32). The progression of arterial rigidity and the consequent loss of arterial elasticity accelerate vascular aging, ultimately lead to renal impairment. Given that arterial rigidity (one of the manifestations of the deterioration of blood vessel walls) is a key etiological factor in RVO, it follows that individuals with CKD are at a higher risk for developing RVO (33). In addition to arteriosclerosis, hypercoagulability stands as another critical factor (34). Diminished renal function has been shown to correlate with increased levels of inflammatory markers, hypercoagulability, abnormal apolipoprotein, elevated plasma Hcy, endothelial dysfunction, and anemia (35), all of which increase the risk of a prothrombotic state and thrombus formation. The suppression of anticoagulant proteins and disruption of fibrinolysis further contribute to the hypercoagulability observed in CKD patients. Consistent with this, elevated levels of prothrombin fragment, thrombin-antithrombin complexes, and plasma Hcy have been identified as risk factors for RVO (36, 37). Kidney serves as the central hub for Hcy metabolism, and the accumulation of Hcy being a key biochemical driver in the progression of CKD (38–40). Hcy affects renal health and accelerates kidney function deterioration through various mechanisms, including impairing vascular endothelial function, intensifying inflammatory reactions, disrupting the vasodilatory effects of nitric oxide, and stimulating matrix metalloproteinase activity (41).

A multitude of studies have confirmed the role of CKD as a risk factor for RVO (42). The prevalence of CKD exhibits considerable variation across different nations, reflecting differences in varying demographic, ethnic, and economic backgrounds (43). According to Dodson et al. (44), among patients with RVO, those of Indian origin have the highest prevalence of CKD (8%), followed by European patients (4.6%) and Asian patients (4%). The incidence of RVO itself also differs by country. The Singapore Eye Disease Epidemiology Study offered insights into the prevalence of RVO among a diverse Asian demographic (45). It was observed that the prevalence was most pronounced among the Chinese population (0.78%), followed by Indians (0.69%) and Malays (0.67%), culminating in an aggregate prevalence of 0.72%. These data provide a foundation for developing targeted RVO prevention strategies in different countries.

Therefore, the East Asian region should be vigilant regarding factors that influence hypercoagulability, arteriosclerosis, and other contributors to CKD. Regular renal function monitoring is a crucial measure for preventing CKD and reducing RVO incidence.

Hypertension is currently recognized as the second most prevalent systemic risk factor for RVO. Multiple studies included in this review have demonstrated that hypertension is also a primary risk factor for RVO in East Asia. The potential mechanisms underlying the increased risk of RVO due to hypertension in East Asia are as follows: Hypertension primarily leads to systemic arteriole lesions. In the early stages, these lesions manifest as fibrosis and hyaline degeneration of the small arterial walls. When hypertension is poorly controlled, it can further lead to arterial spasm and thinning. If hypertension persists over the long term, it can progress to widespread small arteriolar sclerosis in the middle and late stages. The arteries and veins are enclosed within the same vascular sheath. Chronic hypertension can lead to the retinal arterial sclerosis, which compresses the underlying veins, causing venous stasis, slowed blood flow, and an increased risk of thrombosis, ultimately resulting in RVO (6, 46).

Since hypertension is a modifiable risk factor for RVO in East Asia, controlling it can mitigate the risk of this ocular vascular condition. Factors such as high population density and rapid work pace contribute to increased life and work pressures, which may indirectly elevate blood pressure levels. Excessive sodium intake, associated with fluid retention and elevated blood pressure, is also a notable characteristic of hypertension in Asia (47, 48). Moreover, with societal development, environmental pollution has become increasingly severe, contributing to higher blood pressure levels (49). Studies assessing PM2.5 in 79 countries between 1990 to 2013 found that an increase in global population-weighted PM2.5 of 20.4% was found in South Asia, South-East Asia, and China (50).

The aforementioned findings underscore the importance for individuals in East Asia to recognize the risks associated with high-sodium diets and environmental air pollution, and to seek strategies to mitigate these risks. To further address these issues, the policymakers and public health practitioners ought to promote home blood pressure monitoring and regular fundus examination among hypertensive individuals to reduce the risk of RVO.

Hyperlipidemia characterized by an imbalance of cholesterol levels, including low-density lipoprotein cholesterol (LDL-C) and high-density lipoprotein cholesterol (HDL-C) in the blood, is identified as the third major risk factor in this article.

The biological mechanisms linking hyperlipidemia and RVO are complex. Elevated lipid levels can increase plasma viscosity and reduce the surface charge of red blood cells, resulting in enhanced cellular aggregation. Normally, red blood cells carry a negative charge, allowing suspension in the blood; however, elevated lipid levels can neutralize this charge, leading to cellular clumping and adhesion to the vessel walls. Combined with elevated lipoprotein levels, these changes increase blood viscosity and resistance, thereby contributing to the pathogenesis of RVO. High levels of triglycerides (TG), LDL-C, and free fatty acids can induce blood vessels rigidity and loss of elasticity, particularly in small vessels. This degeneration can exacerbate platelet aggregation and alter the plasma membrane composition of red blood cells, causing structural deformation and a subsequent reduction in their oxygen-carrying capacity. Consequently, slowed retinal microcirculation promotes blood stasis and clot formation, potentially obstructing retinal veins and leading to RVO (5).

Global trends in plasma total cholesterol (TC) and non-HDL-C levels have remained relatively stable in recent years. This stability reflects declines in developed countries that previously had high prevalence rates, such as those in Western Europe and Singapore, and increases in developing countries in Southeast Asia and Oceania, attributed to economic growth and lifestyle shifts (51). The reduction in non-HDL-C levels in Western nations is largely due to the widespread use of lipid-lowering medications, particularly statins, and a shift towards diets rich in unsaturated fats. Conversely, the rising prevalence in low- and middle-income countries is due to increased consumption of animal-derived foods and refined carbohydrates, coupled with limited access to lipid-lowering medications. This dichotomy underscores the importance of addressing hyperlipidemia as a risk factor for RVO in East Asia. It highlights the need for dietary interventions to reduce the intake of saturated fatty acids and refined carbohydrates and emphasizes the importance of broader access to and education on lipid-lowering therapies.

This study confirms that DM is an significant risk factor for RVO in the East Asian population. As is well known, DM patients often exhibit high blood sugar levels. Prolonged hyperglycemia can damage vascular endothelial cells, leading to increased vascular permeability (52). Consequently, blood components are more likely to leak out and form thrombi within the blood vessels, thus obstructing the retinal veins. Moreover, changes in the hemorheology of diabetic patients, such as reduced red blood cell deformability and increased platelet aggregation, impair blood mobility, elevate the risk of microangiopathy, and consequently affect retinal venous blood flow, potentially leading to RVO (53). Additionally, DM can trigger inflammation, with the activation of inflammatory factors potentially damaging the vascular endothelium and exacerbating vascular damage (54).

In East Asia, factors such as high population density and rapid work pace contribute to increased life and work pressure. These social and environmental factors may indirectly elevate the incidence of RVO by affecting blood glucose levels. In addition to this, dietary habits, lifestyle and genetic factors in East Asia may also contribute to the higher prevalence of DM. Studies indicate that Asia is home to 60% of the world’s DM patients, with 50% of these from East Asia (55). This correlation may be attributed to lifestyle factors, including high-carbohydrate diet (e.g., rice noodles) and a sedentary lifestyle, which are prevalent in East Asian countries, increasing the prevalence of DM and the risk of RVO (56). Therefore, for patients with DM in East Asia, it is crucial to enhance blood glucose control and rigorously manage cardiovascular risk factors. Routine ophthalmic fundus screenings are essential for the early detection and management of retinal vascular irregularities, which can help in reduce the prevalence of RVO.

Hcy is a sulfur-containing amino acid, it is an intermediate product of methionine metabolism in the body. This study confirms that elevated Hcy level is a significant risk factor for RVO in East Asia. The serum Hcy concentration is typically low in healthy individuals, but factors like renal insufficiency, insufficient intake of folate, vitamin B12, vitamin B6, excessive methionine intake, and genetic metabolic disorders can lead to a rapid increase in serum Hcy levels (57). Clinical studies have shown a strong correlation between serum Hcy levels fluctuations and blood vessel diameter (58). Abnormal levels of serum Hcy and blood lipids can create a vicious cycle, exacerbating RVO progression. On the one hand, blood return obstruction facilitates thrombosis in the retinal vein, which in turn leads to platelet activation, fibrinolytic system impairment, vascular endothelial dysfunction, and dyslipidemia, culminating in elevated Hcy release (59). Additionally, elevated Hcy levels may impair retinal vascular endothelial cell function, promote coagulation, accelerate thrombus formation, and exacerbate RVO progression (5). On the other hand, Hcy can bind with low-density lipoprotein in bloodstream, initiating an immune response and phagocytosis by immune cells, which can raise blood lipid levels, decrease cell mobility, and promote atherosclerosis, thereby exacerbating Hcy in return. Furthermore, Hcy levels correlate with folate concentrations and the metabolic activity of related enzymes, which are under the genetic influence of the methylenetetrahydrofolate reductase (MTHFR) and methionine synthase reductase (MTRR) genes. Research has indicated that individuals possessing the MTHFR677TT genotype exhibit Hcy levels that are approximately 25% higher than those with the MTHFR677CC genotype (60). On account of that increased Hcy levels have a significant and direct impact on kidney function, the genetic polymorphisms of MTHFR and MTRR are likely connected with renal function. Studies have discovered (61) a correlation between the MTHFR C677T gene variant and the deterioration of kidney function, with individuals having the TT genotype (32%) facing a greater risk of reduced glomerular filtration rates compared to those with the CC genotype (25%). Furthermore, Hishiba’s research and that of others have demonstrated a significant association between the MTHFR C677T gene variant and the risk of CKD in the Japanese population (62). These findings suggest that the MTHFR C677T gene variant may contribute to the onset and progression of CKD, either directly or indirectly, through its influence on Hcy levels. Consequently, this results in a heightened prevalence of RVO.

In the East Asian population, elevated Hcy levels are attributed to a higher mutation rate of Hcy-related metabolic genes and dietary habits, among other factors. Particularly in China, the prevalence of elevated Hcy is higher, potentially due to genetic background, dietary habits, and lifestyle factors.

For instance, the distribution and frequency of the MTHFR C677T gene polymorphism vary significantly by country and ethnicity, with the TT genotype prevalence in the Chinese Han population estimated at about 25%, increasing gradually from south to north (63). Poor lifestyle choices, such as alcohol consumption and smoking may also contribute to excessive consumption or inadequate intake of folic acid, vitamin B6, and vitamin B12, resulting in elevated blood Hcy levels (64). Prevention and management strategies for this population should include a proper diet, supplementation with essential vitamins and minerals, regular physical activity, and ongoing monitoring and control of Hcy levels.

In addition to the above four risk factors, this meta-analysis also found atherosclerosis of the carotid artery [OR=2.43, 95%CI: (1.20, 4.93)], NLR [OR=0.35, 95%CI: (0.26, 0.44)], CLD [OR=1.03, 95%CI: (1.13, 1.49)], PLR [OR=23.50, 95%CI: (8.70, 38.30)], age (> 60 years old) [OR=4. 07, 95%CI: (2.11, 6.02)], apolipoprotein A [OR=118.36, 95%CI: (83.52, 153.20)] may be risk factors for RVO; axial length [OR=-2.38, 95%CI: (-3.20, -1.55)], folic acid [OR=-3.69, 95%CI: (-5.18, -2.21)] may be a protective factor for RVO. As these nine RVO-related factors have been included in too few studies (≤2 papers), with high heterogeneity (*I^2^* ≥50%) or *P* > 0.05, further studies are needed to confirm their correlation with RVO. The main risk (or protection) factors related to RVO have not been included in this paper for the time being.

A global meta-analysis of risk factors for RVO launched in 2019 found that hypertension, history of heart attack, history of stroke, elevated total cholesterol levels, and elevated creatinine levels were global risk factors for RVO (65). Compared with this study, abnormal renal indicators such as hypertension, hyperlipidemia, and elevated creatinine levels are also risk factors for RVO in the East Asian population. However, in East Asian populations, particularly in South Korea, CKD is the leading risk factor for RVO. At the same time, the risk factors of RVO in East Asian populations include DM and high Hcy, which provides a more accurate prevention and treatment direction with more population and regional characteristics for RVO prevention and treatment in East Asian populations.

The limitations of the present study should be also clarified. First, all the findings in this study are related to systemic risk factors for RVO, and there is a lack of studies on local ocular risk factors. Second, the research articles included in this paper are highly heterogeneous, and the lack of relevant subgroup analysis such as age and gender may have a certain impact on the comprehensiveness of the research results. Third, the research articles on RVO in China included in this paper have regional limitations, and there is a lack of national research on RVO risk factors, and there are relatively few relevant studies in Japan and South Korea. Fourth, the lack of relevant data in the article was eliminated in this paper, which may lead to certain deviations in the research results. In the future, data can be further collected from the author of the original text, and statistics can be conducted again to correct the deviations. Fifth, there is a lag in the publication of some journals, and this study may miss some literature. To enhance the thoroughness of identifying risk factors associated with RVO, more inclusive screening criteria will be implemented, and sensitivity analyses will be performed to validate the findings. These limitations should be addressed in subsequent studies.

## 5 Conclusion

This meta-analysis establishes a significant association between the incidence of RVO and several identified risk factors. Conditions such as CKD, hypertension, hyperlipidemia, DM, high Hcy levels are recognized as major contributors to RVO development in the East Asian population. This research indicates that dietary management may serve as a pivotal strategy in the prevention and management of RVO among East Asian populations. Adopting a dietary framework that emphasizes low sodium, low fat, and minimal refined carbohydrates, alongside a balanced and moderate eating pattern, coupled with targeted supplementation of folic acid, vitamin B6, and vitamin B12, can facilitate the integration of RVO prevention into the daily lifestyle of East Asians. It is crucial for ophthalmologists to carefully consider these risk factors in the diagnostic and therapeutic process of RVO. By doing so, they can improve preventive strategies and provide more targeted and comprehensive care to patients.

## Data Availability

The raw data supporting the conclusions of this article will be made available by the authors, without undue reservation.
